# Supplementary material for: A Transcriptomics-Based Meta-Analysis Combined With Machine Learning Identifies a Secretory Biomarker Panel for Diagnosis of Pancreatic Adenocarcinoma
Source: Front Genet. 2020 Sep 10;11:572284. doi: 10.3389/fgene.2020.572284 (PMC7511758; doi:10.3389/fgene.2020.572284)
Supplement: FIGURE S1 — Pathway enrichment analysis of the 74 PDAC-specific secretory genes. [file Data_Sheet_1.pdf]

Supplementary Figure S1. Pathway enrichment analysis of the 74 PDAC-specific secretory genes.

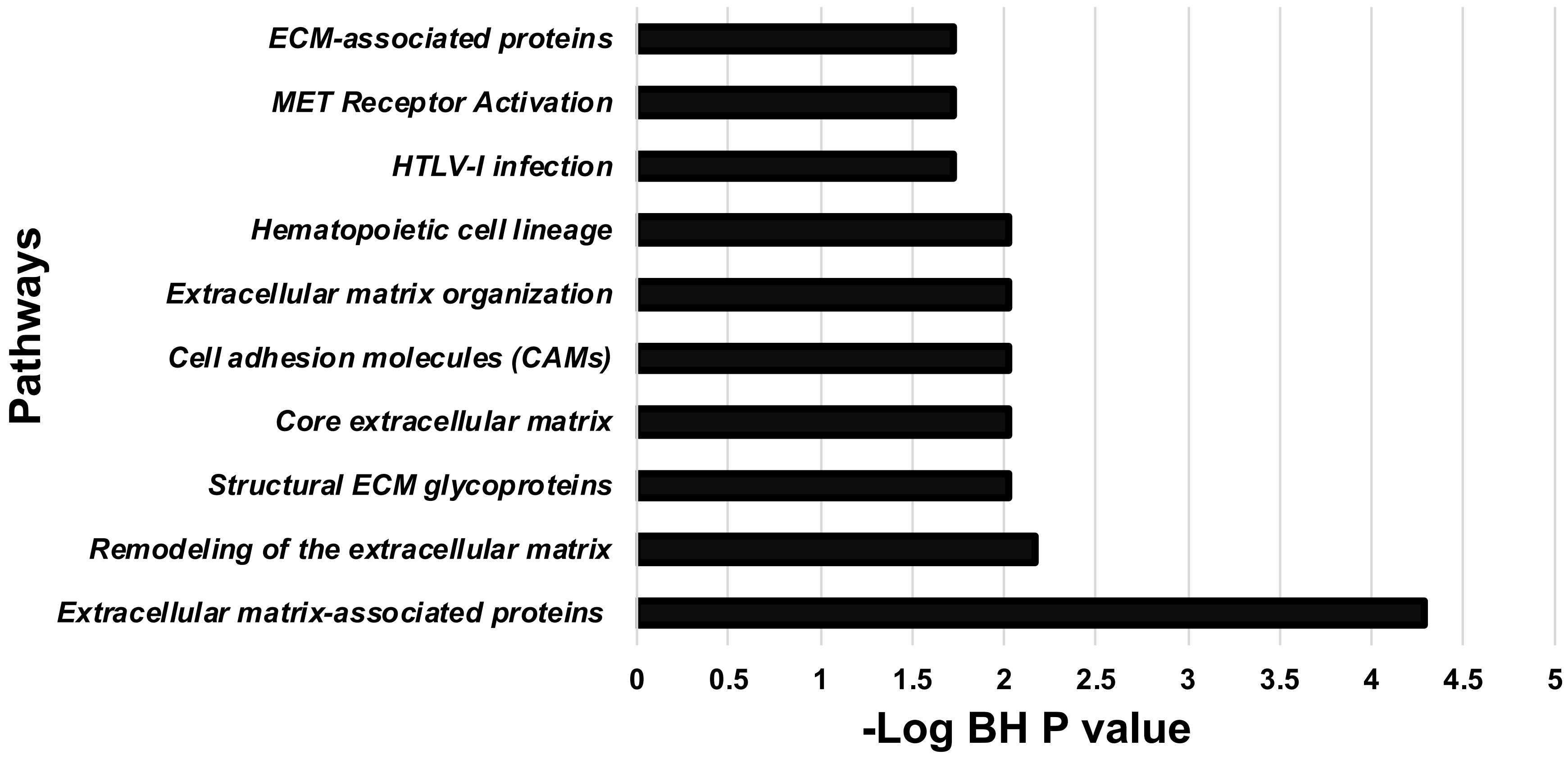

Supplementary Figure S2: Upregulated Secretory genes in training datasets.

A) Heatmap of 27 upregulated secretory genes in PDAC for two of the three tissues and one of the two blood datasets. **B)** PCA plots for each training datasets using 27 upregulated secretory genes.

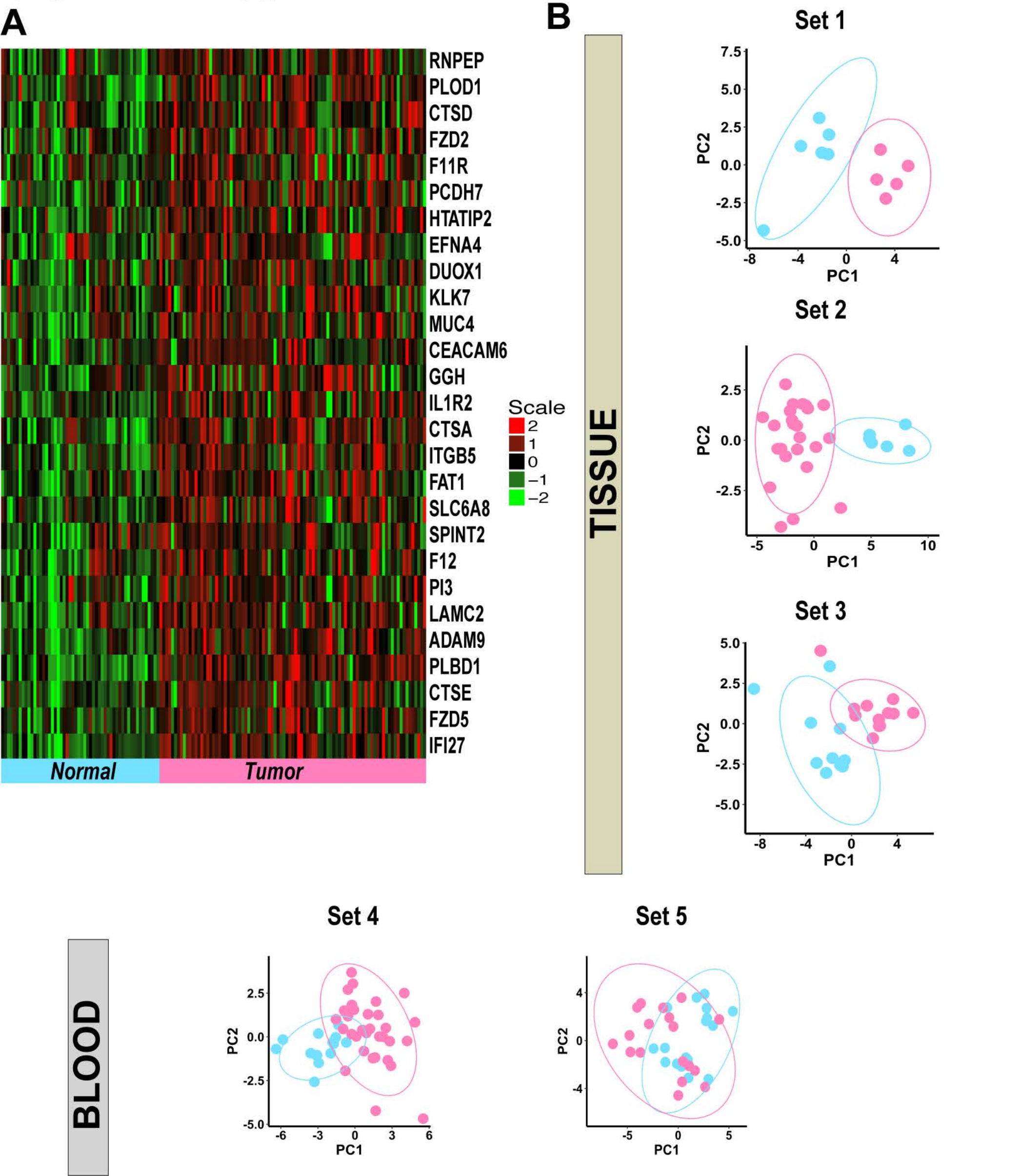

Supplementary Figure S3: Performance of 9-gene PDAC classifier on training sets using leave one out cross-validation (LOOCV). A) Diagnostic performance of the 9-gene PDAC classifier on the five training sets. Sensitivity (Sens) and Specificity (Spec) are indicated for each dataset. **B)** AUC plot for 9-gene PDAC classifier on the three tissue training datasets. **C)** AUC plot for 9-gene PDAC classifier on the two blood training datasets.

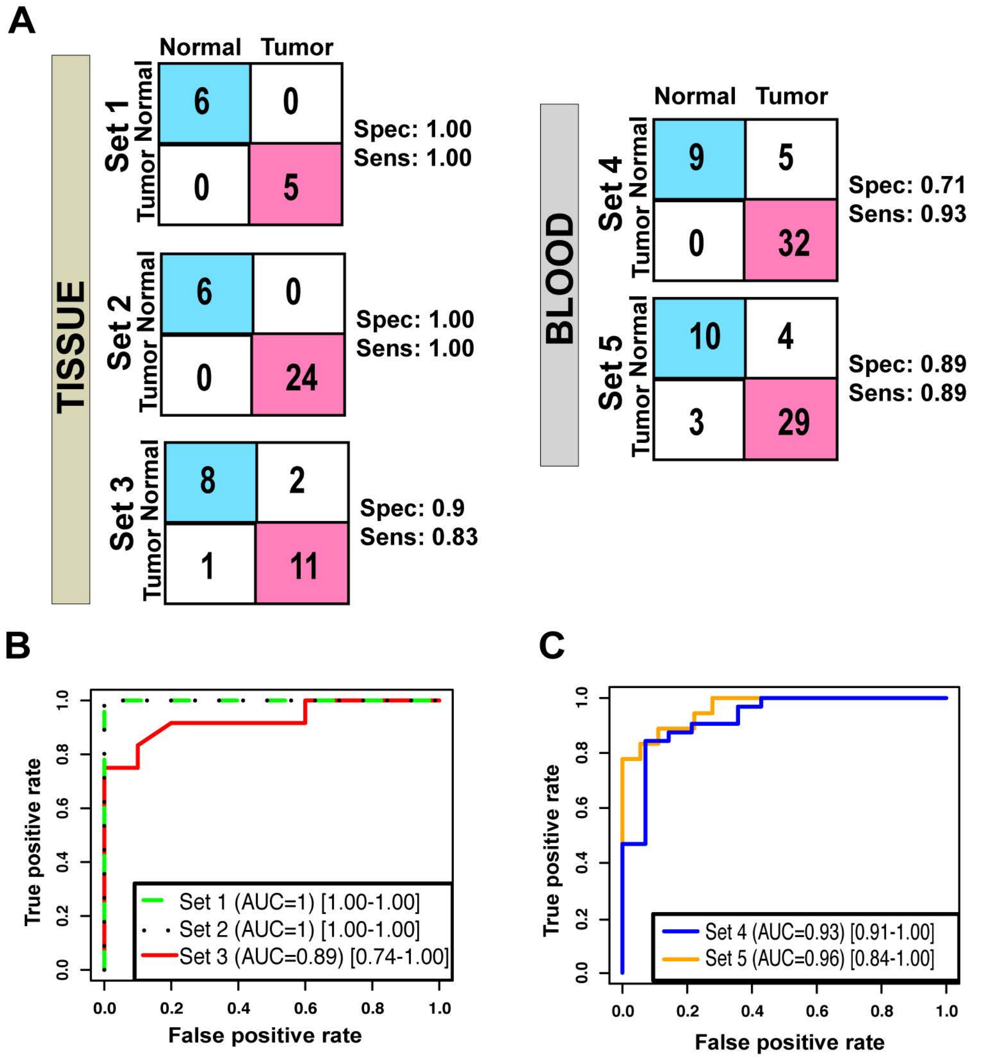

Supplementary Figure S4: The metrics for training datasets using the 9-biomarker panel genes. A) Boxplot of the averaged expression of the genes across all the five training datasets. **B)** PCA plots for each training datasets using the 9-biomarker panel genes.

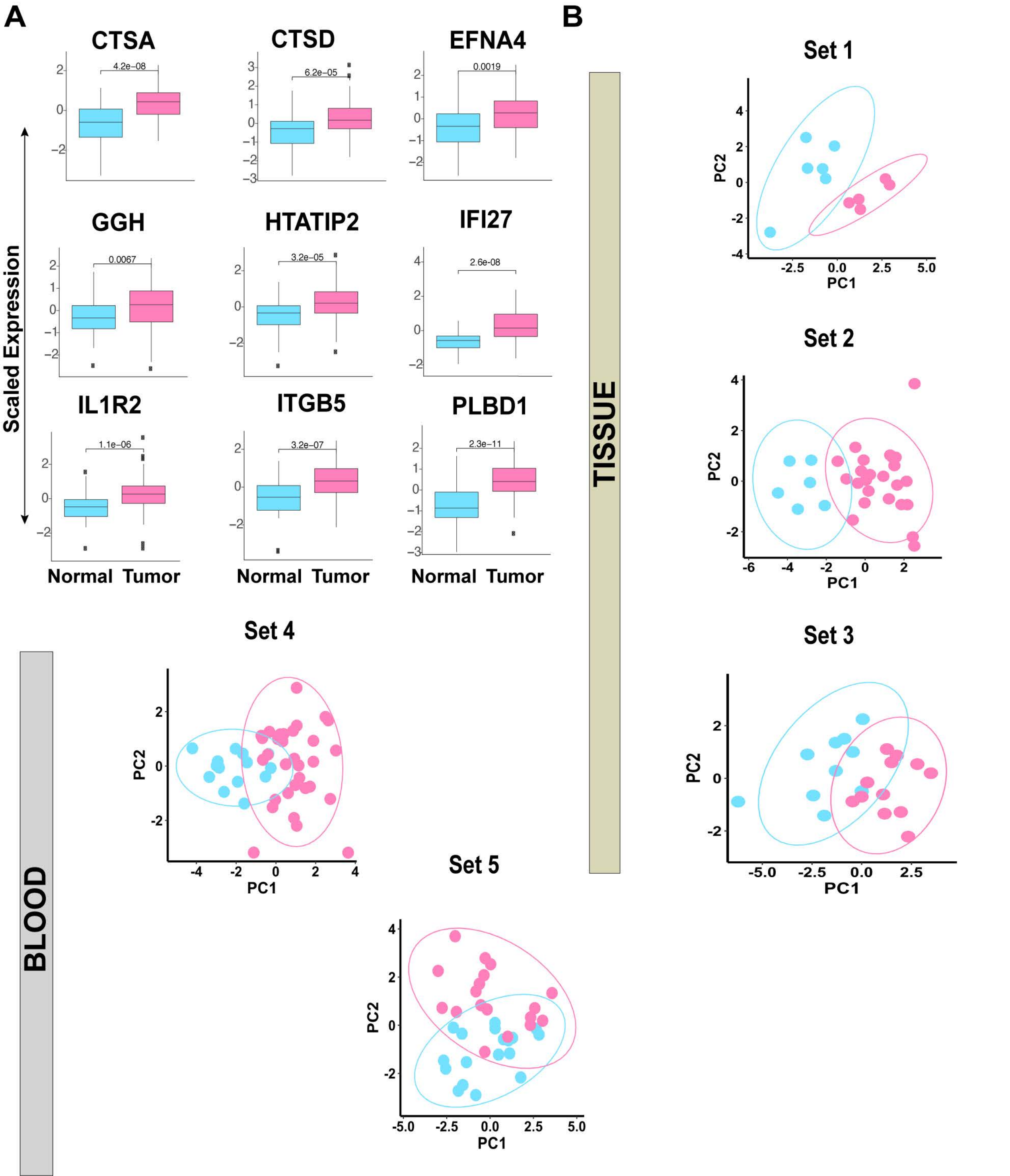

Supplementary Figure S5: The assessment metrics for testing datasets using the 9-biomarker panel genes. A) Heatmap of the 9 PDAC-upregulated marker genes. **B)** PCA plots in six independent testing datasets..

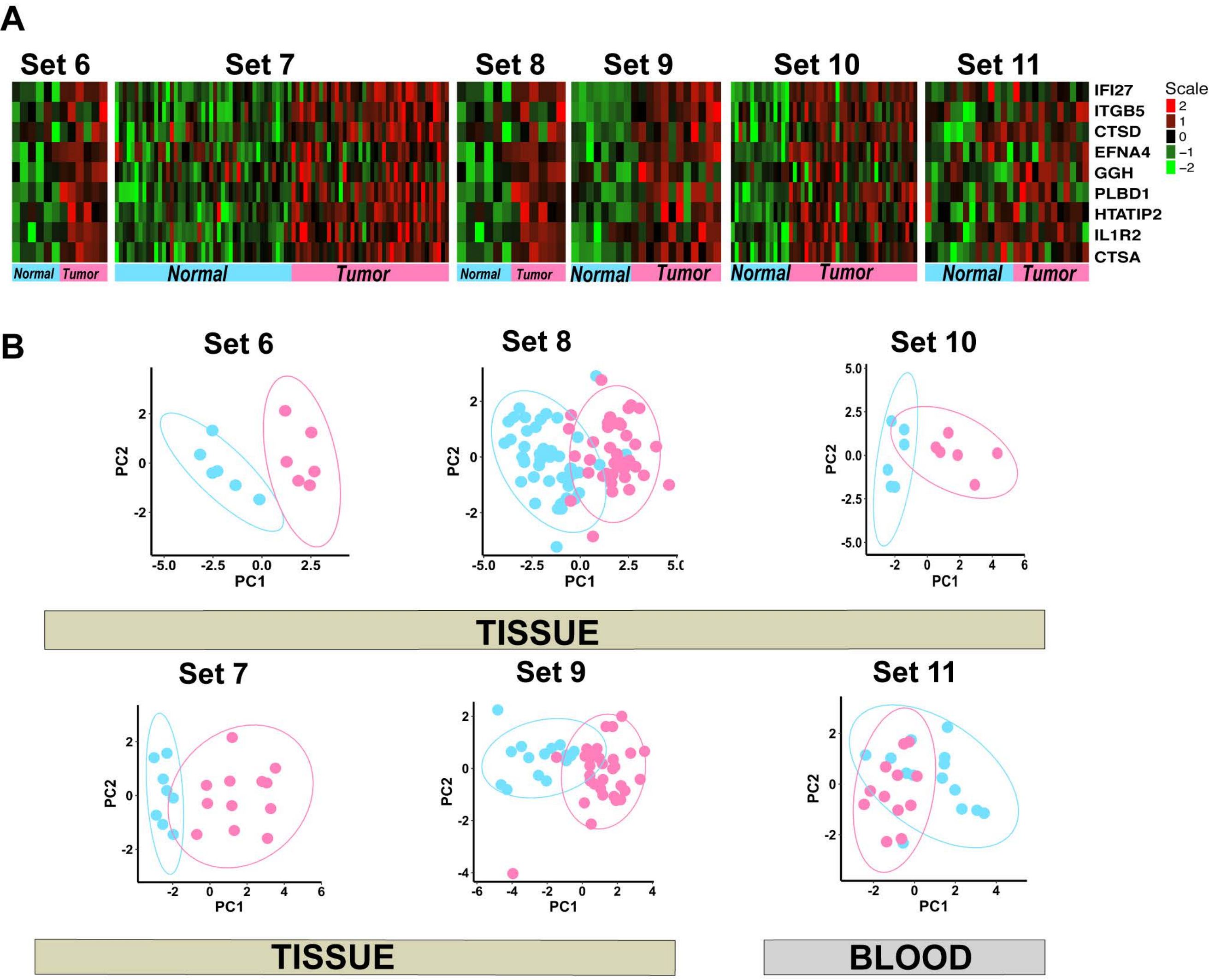

Supplementary Figure S6: The assessment metrics for validation datasets using the 9-biomarker panel genes. Heatmaps **(A)** and PCA plots **(B)** based on biomarker panel genes in validation sets.

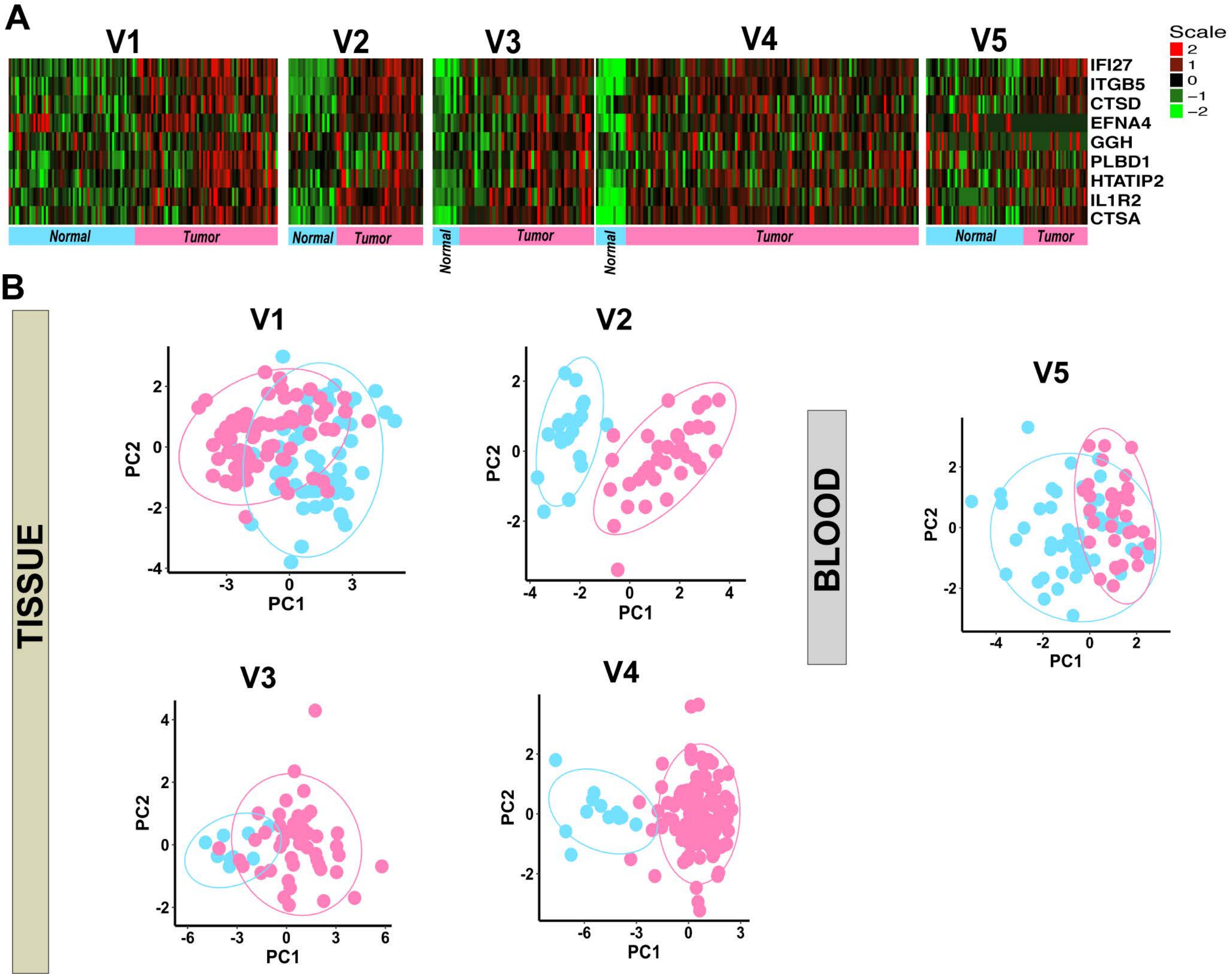

Supplementary Figure S7: The assessment metrics for PV1-3 dataset using the 9-biomarker panel genes. A) PCA plots of three different prospective validation datasets. **B)** Heatmaps of the 9-marker genes panel. **C)** Boxplots of the expression of the genes.

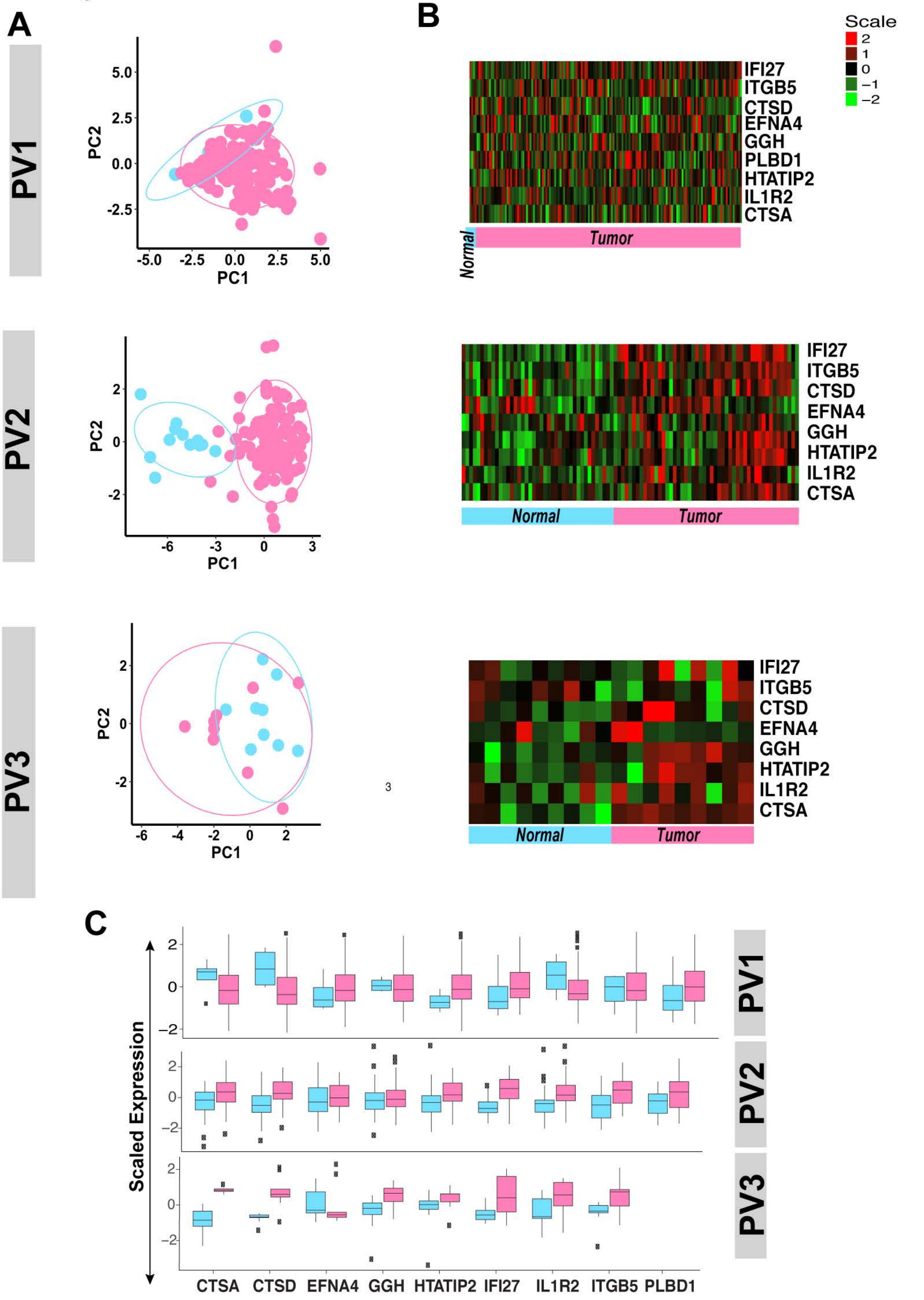

Supplementary Figure S8: Survival curve of 9-gene-based PDAC classifier and combined genes.

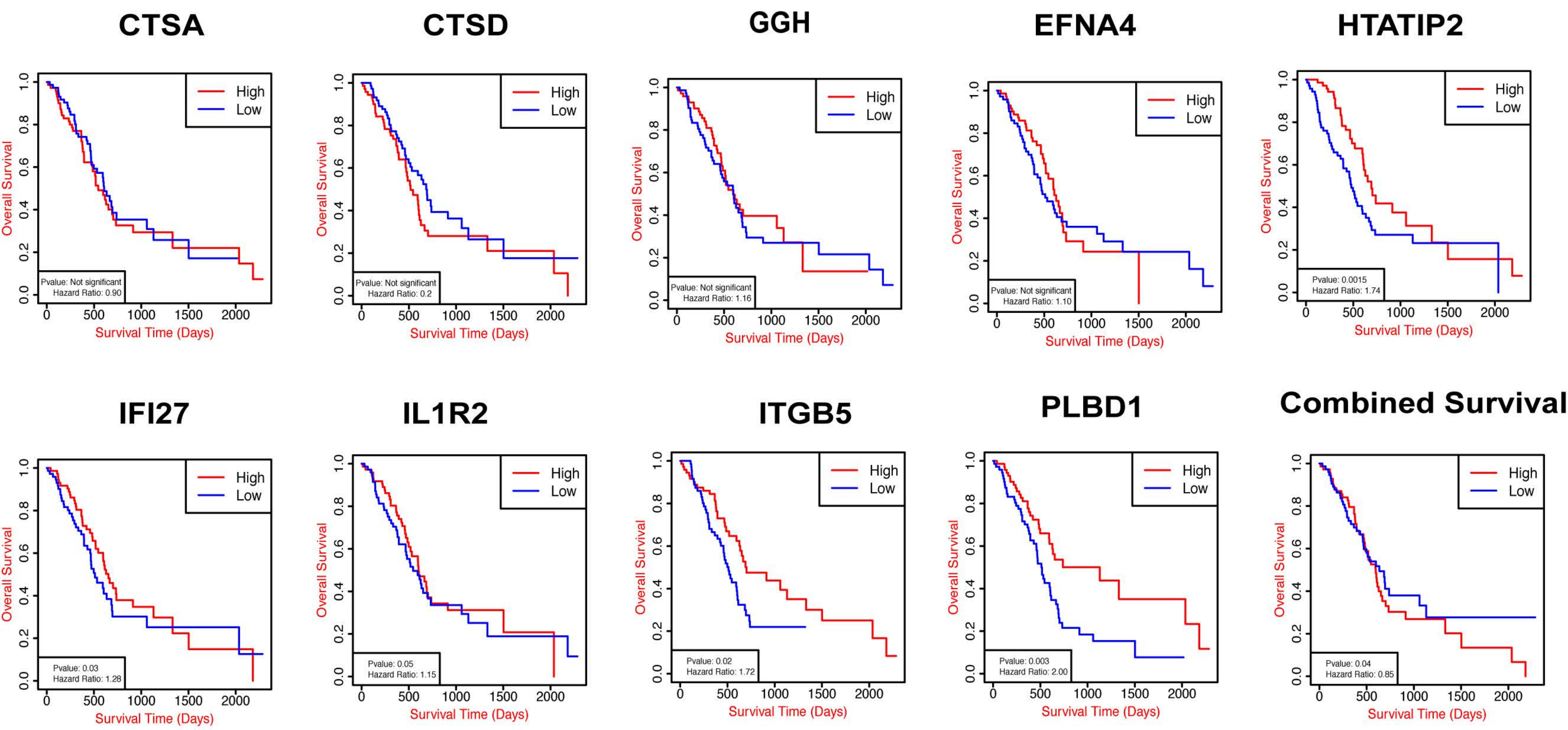

Supplementary Figure S9: The assessment metrics for PV4 dataset using the 9-biomarker panel genes. A) PCA plots for precursor lesions in three stages IPMA, IPMN and IPMC. **B)** Heatmaps of the 9-marker genes panel. **C)** Boxplots of the expression of the genes in precursor lesions.

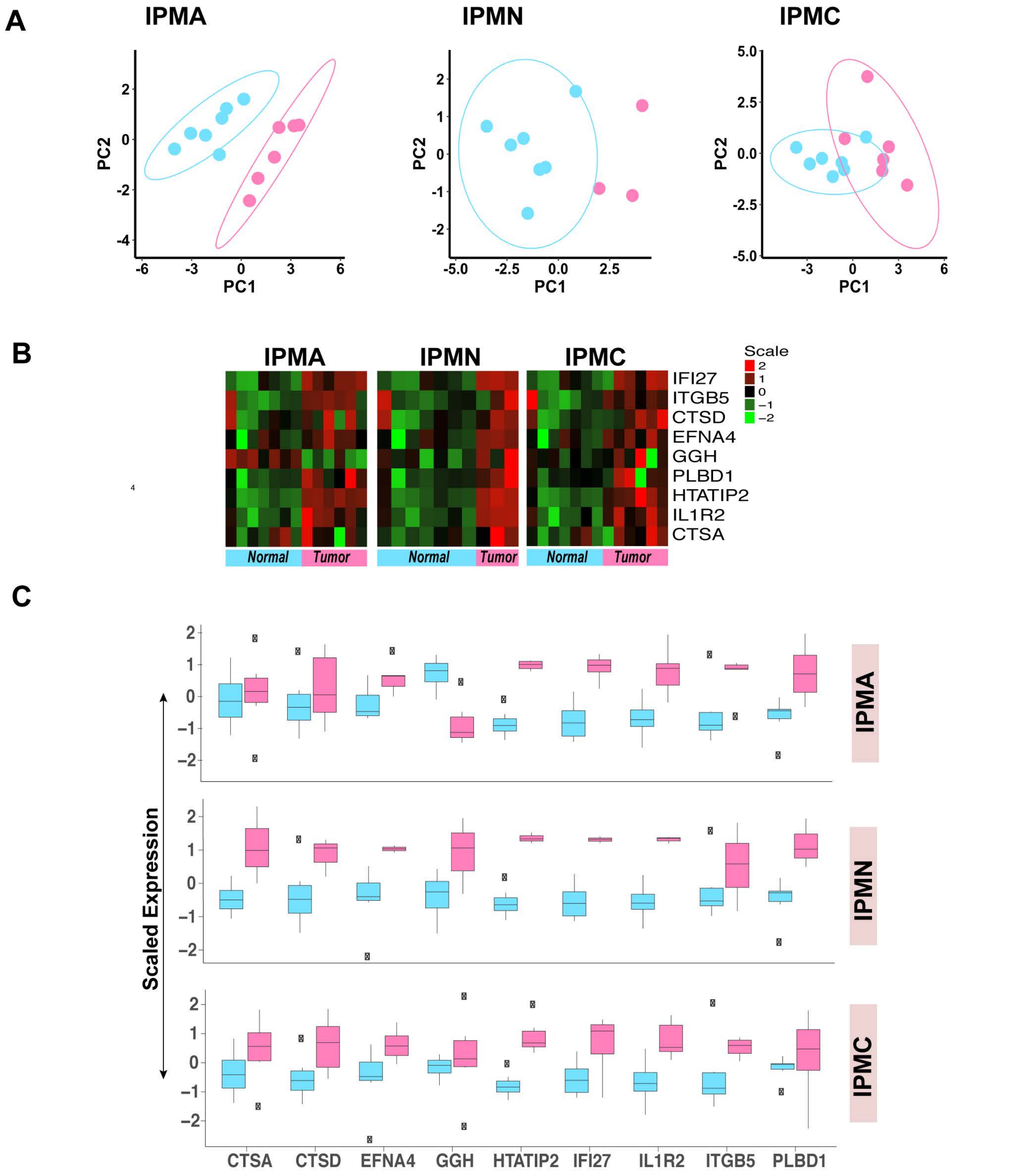

Supplementary Figure S10: Comparative perfromance of 9-gene-based PDAC classifier with different previously established biomarkers. AUC plot for 9-gene-based PDAC classifier across the training and validation datasets. The measures of performances e.g. accuracy, sensitivity, specificity and AUC are mentioned in **Supplementary table 4**.

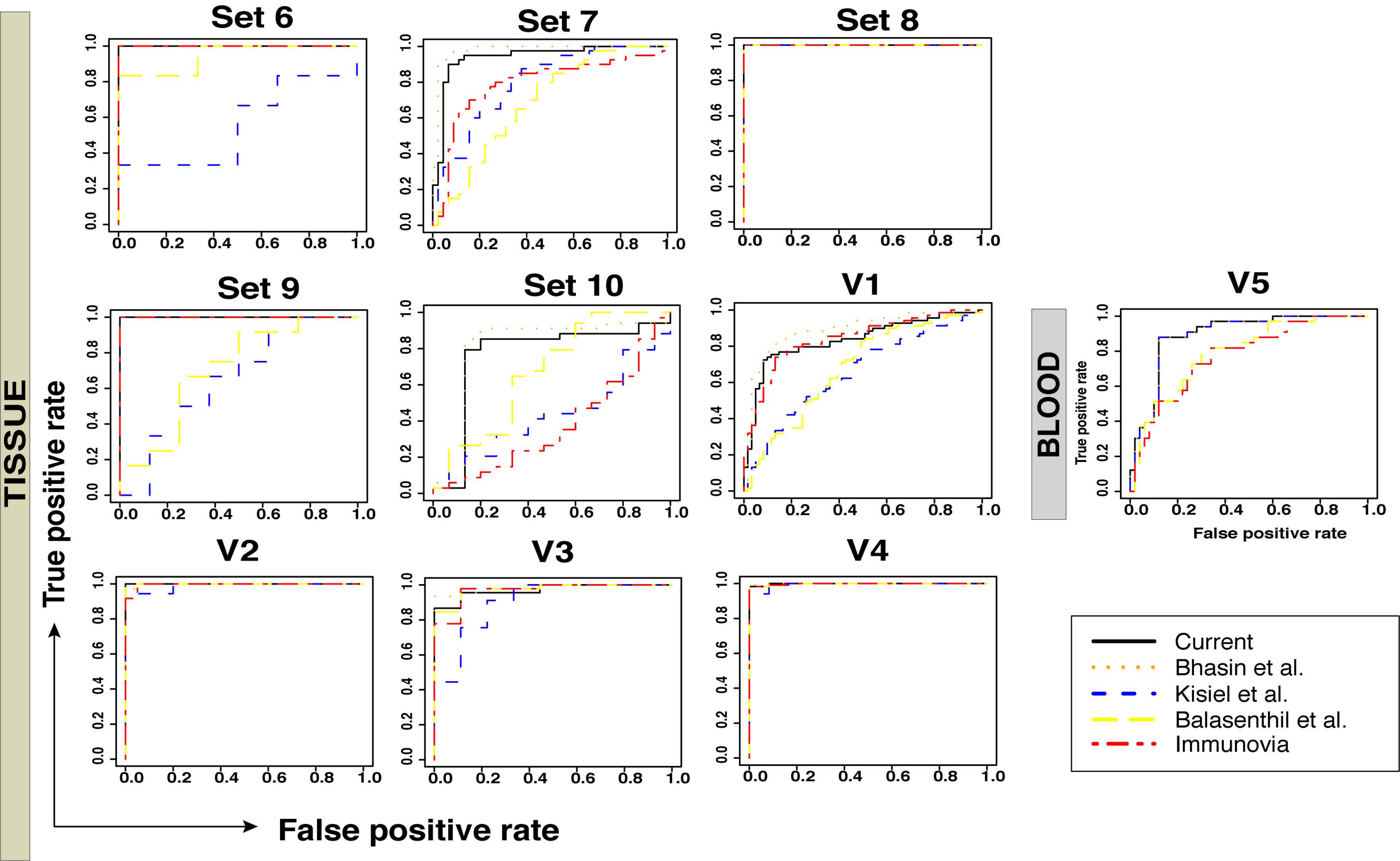

Supplementary Figure S11: Expression of 9-gene markers in different pancreas cell-types in both healthy and tumor states. The expression of these genes is high in tumor state (CTSA, CTSD, EFNA4, GGH, HTATIP2, IFI27 and ITGB5) or they are not expressed at all in healthy state (IL1R2 and PLBD1) Source: Peng J et al., Cell Research, 2019 [9] . This is also consistent with protein expression of the genes as measured by antibody staining experiments by Human protein atlas.

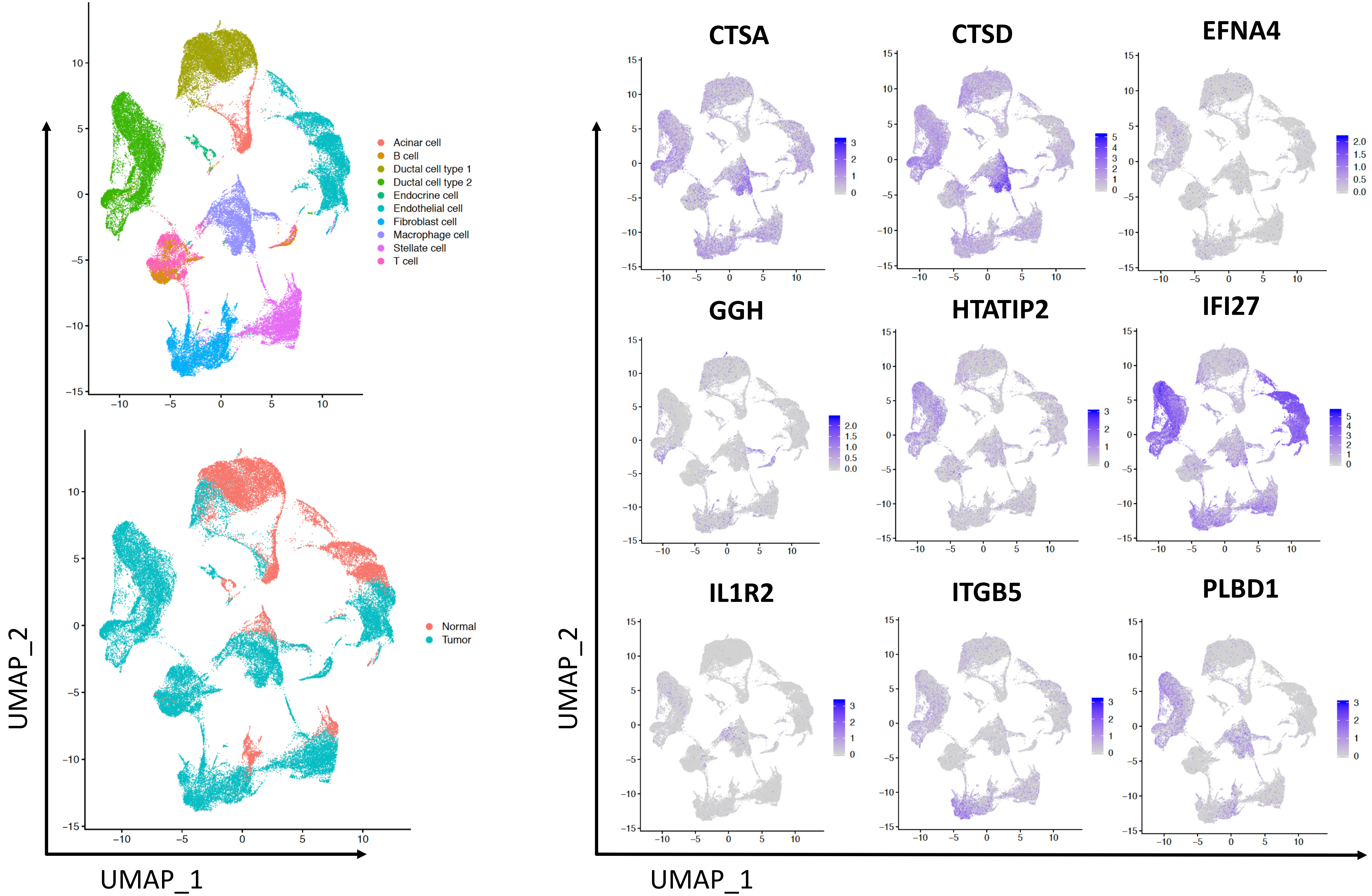

Supplementary Figure S12: Immunolabeling of protein expression of nine genes selected for the classifier in pancreatic cancer. Light blue is low staining; blue is moderate staining and brown is high.

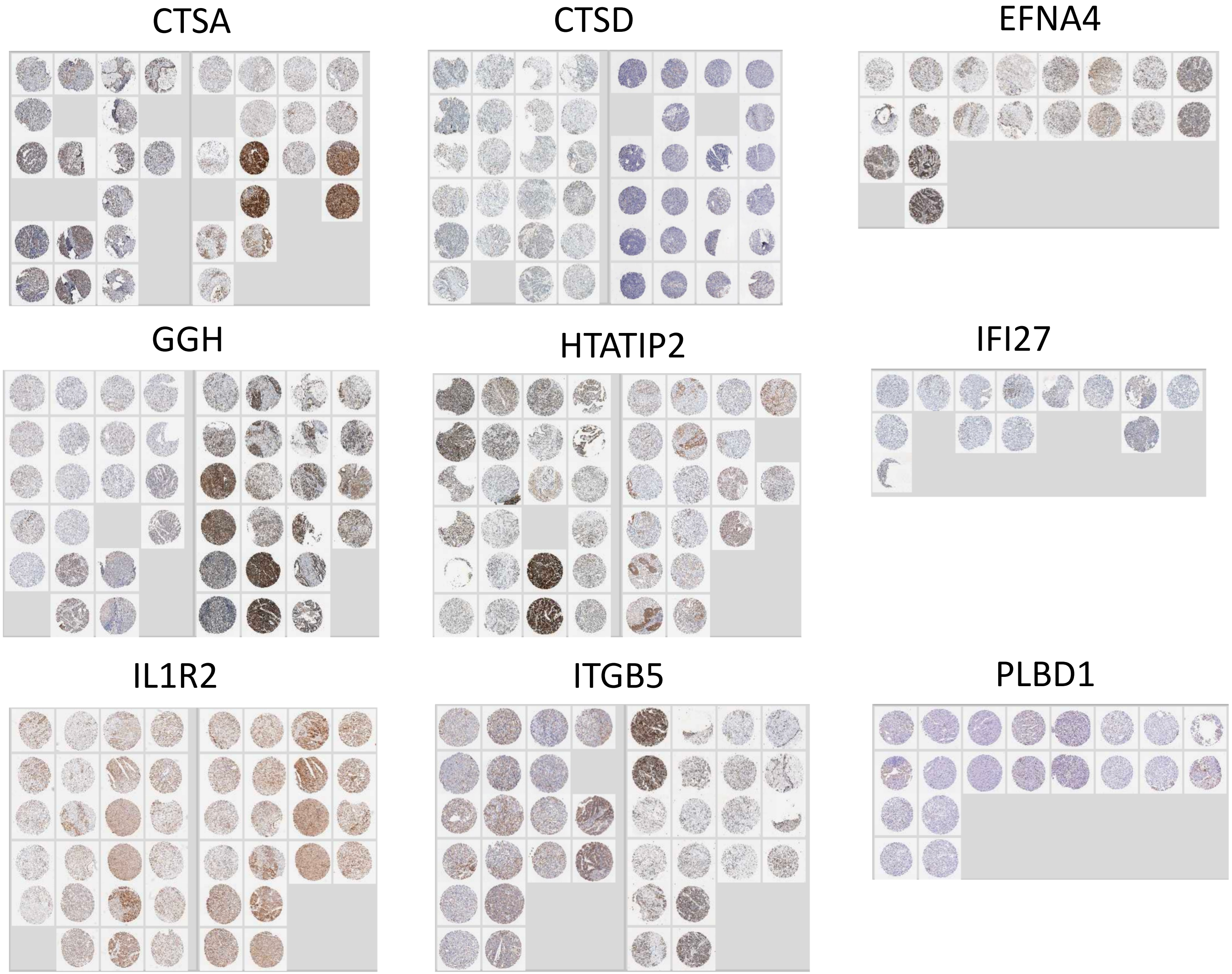

Table S1. Log2 fold change of the significantly differentially Expressed genes identified from different training datasets.

	Tissue datasets			Blood datasets	
**Gene Symbols**	**Set 1**	**Set 2**	**Set 3**	**Set 4**	**Set 5**
DNASE1L3	NA	-0.0102308	-0.9566372	-1.6733822	-1.7714724
LRRN3	-1.2863319	NA	-0.8128004	-1.4166422	-1.5215287
SATB1	-0.6442892	NA	-1.2565995	-1.1935436	-0.9054894
PTGDS	NA	-0.0572214	-1.5519188	NA	-2.5739691
EBI3	NA	-0.0062389	NA	-1.4540712	-2.302169
GZMK	NA	-0.1240268	NA	-1.4072116	-2.2390033
CTSW	-0.4297419	-0.0473606	-1.930786	NA	-2.2255588
FCMR	-0.9812385	-0.1511	NA	-0.7727684	-1.7854954
CD79A	-0.9141881	NA	-1.082996	NA	-1.5609809
GDF10	NA	-0.0063355	-1.4747534	NA	-1.5383126
CD22	-0.8527311	-0.0147575	-1.2656263	NA	-1.4138414
CD27	-0.5652415	-0.1470219	NA	-0.6619055	-1.4120213
IL12RB2	-0.3853798	NA	NA	-0.9444184	-1.3933073
CD160	NA	-0.0872521	NA	-1.2649517	-1.3814509
COCH	NA	-0.010724	-1.5067794	NA	-1.3124335
NELL2	-0.9708002	-0.0632795	-0.8513571	NA	-1.2611376
SLAMF1	-0.4658339	-0.0380011	-0.8521605	NA	-1.1877711
HLA-DPB1	NA	-0.0203238	NA	-0.8826801	-1.1652361
CD3E	-0.4338554	-0.1082692	-0.7280421	NA	-1.1291937
NLGN4X	NA	-0.0069318	-1.5524494	NA	-1.1164799
DNAJB9	NA	-0.0167893	NA	-0.7593761	-1.0819126
IL2RB	-0.7635839	-0.1381649	-0.7403784	NA	-1.0255142
CRY2	-0.2696785	NA	-1.2989376	NA	-0.973885
PARM1	-0.4026864	-0.0082114	NA	-1.4185854	-0.9172337
ACACB	-0.2130128	NA	-0.8688955	NA	-0.8474979
NRCAM	-0.4756645	NA	-0.7238321	NA	-0.7926464
SPOCK2	-0.4748107	-0.0992499	-0.7014596	NA	-0.7785083
EIF2AK3	-0.4004008	-0.0219009	NA	-0.5540014	-0.7118028

SMARCA2	-0.2730799	-0.0460831	NA	-0.8401909	-0.6641317
PRNP	NA	-0.0618262	-0.5526453	NA	-0.4949372
SARAF	NA	-0.1104759	NA	-0.4872521	-0.4830139
ASIP	NA	-0.0089826	-2.1325008	-2.2481357	NA
CD226	NA	-0.0155533	-0.7242504	-1.8787667	NA
FZD3	-0.264414	-0.0079939	-1.3388057	-1.5341284	NA
FAM171A1	NA	-0.0244005	-0.9119432	-0.9800952	NA
RNPEP	NA	0.06499014	0.95398998	NA	0.41768265
PLOD1	NA	0.09547286	NA	0.92561755	0.50155107
SLC10A3	NA	0.01763271	NA	0.74658099	0.50682203
CTSD	NA	0.04746329	1.2754561	NA	0.76021333
FZD2	NA	0.02164895	1.44884731	NA	0.81139532
F11R	NA	0.01195715	NA	0.95747149	0.83218316
MET	NA	0.01887576	NA	0.89777193	0.85066331
PCDH7	0.22264695	NA	NA	1.36629866	1.01153058
HTATIP2	NA	0.02361367	0.70979447	NA	1.02897248
ECM1	NA	0.01714136	NA	1.17384734	1.18387031
NDNF	0.33029215	NA	NA	1.48913214	1.25959925
TINAGL1	NA	0.00767782	NA	1.35358756	1.3607891
EFNA4	NA	0.01499515	1.54675037	NA	1.53163682
TMEM158	NA	0.11370092	NA	1.93498007	1.63762385
DMBT1	0.20609413	NA	NA	2.37426481	1.68706202
CA9	NA	0.00649849	NA	2.23365804	1.699295
DUOX1	NA	0.00887676	NA	2.44828895	2.01800441
KLK7	NA	0.00652333	NA	4.27690315	2.61510498
TFF3	NA	0.02998763	NA	1.36976068	3.02308923
MUC4	NA	0.01660057	NA	4.34028652	4.77504924
CEACAM6	0.68734494	NA	NA	1.84579246	5.37084254
MICB	NA	0.0454302	1.12708641	0.61441869	NA
GGH	0.40428577	NA	1.16431707	0.64016283	NA
IL1R2	NA	0.02805492	1.96252861	1.19676844	NA
CTSA	NA	0.06486968	1.12882668	0.569448	0.56228617

ITGB5	0.40532311	NA	0.56996513	0.86378621	0.89056218
CD55	0.43910958	NA	1.68442144	1.38634247	1.18032619
FAT1	NA	0.00801813	1.05838548	1.09973351	1.34356191
SLC6A8	NA	0.07658205	0.88715672	2.45194083	1.69464796
SPINT2	0.21089938	NA	1.52394086	1.45628448	1.81526649
F12	NA	0.01065864	1.57281184	2.89125329	2.09305047
PI3	NA	0.13098904	1.54565261	3.08918788	2.97440508
LAMC2	NA	0.00581006	1.15880058	2.4392472	3.28863854
ADAM9	0.65589477	0.01143644	1.21182415	NA	1.03384287
PLBD1	0.98046509	0.10857842	1.51463411	NA	1.38322127
CTSE	0.55488335	0.01164965	NA	2.39668584	4.75791587
FZD5	0.17583608	0.00912522	0.88362041	1.10056425	0.74346978
CDCP1	0.17986381	0.01064018	1.35564396	1.10288462	1.45556502
IFI27	0.49426769	0.11556995	2.84247197	2.16446631	1.84500054

Table S2: Direction of differentially upregulated genes validated via boxplot analysis. Upregulated are shown with green background and ones with opposite direction are colored black.

	Tissue datasets			Blood datasets	
	**Set 1**	**Set 2**	**Set 3**	**Set 4**	**Set 5**
RNPEP	Up	Up	Up	Up	Up
PLOD1	Up	Up	Up	Up	Up
CTSD	Up	Up	Up	Up	Up
FZD2	Up	Up	Up	Up	Up
F11R	Up	Up	Up	Up	Up
PCDH7	Up	Up	Up	Up	Up
HTATIP2	Up	Up	Up	Up	Up
EFNA4	Up	Up	Up	Up	Up
DUOX1	Up	Up	Up	Up	Up
KLK7	Up	Up	Up	Up	Up
MUC4	Up	Up	Up	Up	Up
CEACAM6	Up	Up	Up	Up	Up
GGH	Up	Up	Up	Up	Up
IL1R2	Up	Up	Up	Up	Up
CTSA	Up	Up	Up	Up	Up
ITGB5	Up	Up	Up	Up	Up
FAT1	Up	Up	Up	Up	Up
SLC6A8	Up	Up	Up	Up	Up
SPINT2	Up	Up	Up	Up	Up
F12	Up	Up	Up	Up	Up
PI3	Up	Up	Up	Up	Up
LAMC2	Up	Up	Up	Up	Up
ADAM9	Up	Up	Up	Up	Up
PLBD1	Up	Up	Up	Up	Up
CTSE	Up	Up	Up	Up	Up
FZD5	Up	Up	Up	Up	Up
IFI27	Up	Up	Up	Up	Up
SLC10A3	Up	Up	Up		Up
TMEM158	Up	Up	Up		Up
MICB	Up	Up	Up		Up
CD55	Up	Up	Up	Up	
CDCP1	Up	Up	Up	Up	
MET	Up	Up		Up	Up

NDNF	Up	Up		Up	Up
TINAGL1	Up	Up		Up	Up
DMBT1	Up	Up		Up	Up
CA9	Up	Up		Up	Up
TFF3	Up	Up		Up	Up
ECM1	Up	Up		Up	

Table S3: Comparative performance of 9-gene PDAC Classifier with different previously established biomarkers in training, test and validation datasets. Sets with green background are datasets derived from blood. All mustard colored cells have AUC > 0.80 whereas light blue cells indicate low specificity or sensitivity despite of high AUC. For black shaded cells all the genes corresponding to the mentioned studies cannot be identified.

		Current				Bhasin et al				Balasenthil et al				Kisiel et al				Immunovia			
		Acc	Sens	Spec	AUC	Acc	Sens	Spec	AUC	Acc	Sens	Spec	AUC	Acc	Sens	Spec	AUC	Acc	Sens	Spec	AUC
TRAINING	Set 1	1	1	1	1	0.91	1	0.83	1	0.71	0.6	0.83	0.76	0.63	0.6	0.67	0.73	0.8	0.6	1	1
	Set 2	1	1	1	1	1	1	1	1	0.5	1	0	0.57	0.5	1	0	0.07	1	1	1	1
	Set 3	0.87	0.83	0.9	0.89	0.95	1	0.9	0.98	0.47	0.83	0.1	0.16	0.2	0.41	0	0.15	0.78	0.67	0.9	0.92
	Set 4	0.82	0.93	0.71	0.93	0.49	0.97	0	0.12	0.5	1	0	0.01	0.5	1	0	0.35	0.72	0.88	0.57	0.77
	Set 5	0.86	0.89	0.89	0.97	0.5	0.45	0.56	0.53	0.78	0.78	0.78	0.81	0.47	0.44	0.5	0.49	0.59	0.56	0.62	0.64
TEST	Set 6	1	1	1	1	1	1	1	1	0.66	0.83	0.5	0.64	0.91	1	0.83	1	1	1	1	1
	Set 7	0.92	0.9	0.93	0.94	1	1	1	1	0.6	0.9	0.25	0.7	0.88	0.95	0.8	0.86	1	1	1	0.99
	Set 8	1	1	1	1	1	1	1	1	1	1	1	1	1	1	1	1	0.91	1	0.83	1
	Set 9	0.95	0.91	1	1	1	1	1	1	0.9	0.75	1	0.85	0.75	0.37	1	0.93	0.9	0.75	1	1
	Set 10	0.96	0.93	1	0.94	0.93	0.8	1	0.87	0.75	0.2	1	0.71	0.83	0.46	1	0.89	1	1	1	0.92
	Set 11	0.73	0.75	0.71	0.8	0.47	0	0.93	0.22	0.71	0.58	0.85	0.65	0.51	0.16	0.85	0.23	0.29	0.09	0.5	0.18
VALIDATION	V1	0.79	0.76	0.83	0.83	0.84	0.86	0.82	0.92	0.6	0.21	0.95	0.72	0.76	0.62	0.88	0.7	0.89	0.77	1	0.94
	V2	0.98	0.97	1	1	0.98	0.95	1	0.99	1	1	1	1	0.9	0.85	1	0.9	1	1	1	1
	V3	0.94	1	0.89	0.98	0.98	0.88	1	0.99	0.96	0.77	1	0.99	0.96	0.77	1	0.96	0.94	0.66	1	0.96
	V4	0.95	1	0.91	0.99	0.99	0.91	1	1					0.99	0.91	1	0.99				
	V5	0.83	0.84	0.82	0.89					0.67	0.96	0.24	0.82								
